# Musical plus phonological input for young foreign language readers

**DOI:** 10.3389/fpsyg.2015.00286

**Published:** 2015-03-17

**Authors:** M. C. Fonseca-Mora, Pilar Jara-Jiménez, María Gómez-Domínguez

**Affiliations:** ^1^English Language Department, University of HuelvaHuelva, Spain; ^2^Department of Developmental, Educational and Social Psychology and Methodology, Jaume I UniversityCastellón, Spain

**Keywords:** phonological awareness, literacy, foreign language, reading, working memory, music

## Abstract

Based on previous studies showing that phonological awareness is related to reading abilities and that music training improves phonological processing, the aim of the present study was to test for the efficiency of a new method for teaching to read in a foreign language. Specifically, we tested the efficacy of a phonological training program, with and without musical support that aimed at improving early reading skills in 7–8-year-old Spanish children (*n* = 63) learning English as a foreign language. Of interest was also to explore the impact of this training program on working memory and decoding skills. To achieve these goals we tested three groups of children before and after training: a control group, an experimental group with phonological non-musical intervention (active control), and an experimental group with musical intervention. Results clearly point to the beneficial effects of the phonological teaching approach but the further impact of the music support was not demonstrated. Moreover, while children in the music group showed low musical aptitudes before training, they nevertheless performed better than the control group. Therefore, the phonological training program with and without music support seem to have significant effects on early reading skills.

## Introduction

A large amount of literature has been published on reading acquisition difficulties in native (L1) or in second language (L2) learning. Several factors, such as phonological and decoding skills have often been described as variables of crucial importance in the learning-to-read process (Brady, 1991; Melby-Lervåg et al., 2012). In their review, Hulme and Snowling's, (2014) conclude that deficits in oral language skills as well as deficits in phonological language skills and problems in phoneme awareness, letter–sound knowledge and rapid automatized naming are of primary importance to account for learning to read difficulties. Jongejan et al. (2007) also considered that phonological language skills are important for L1 and L2 acquisition as they provide the necessary tools for lexical access and reading. The lack of oral language input in L2 acquisition is problematic when the pronunciation rules of L1 differ from L2. In this context, finding alternative research-based teaching approaches that could help learners to achieve foreign language literacy skills is very relevant.

Several results in the last two decades points to music as an aid in learning to read (Butzlaff, 2000; Bolduc, 2008; Standley, 2008; Lessard and Bolduc, 2011; Toscano-Fuentes and Fonseca-Mora, 2012) but the nature of this connection still needs to be clarified. Ott et al. (2011) suggest that early phonetic processing of verbal or non-verbal stimuli is differently organized depending on musical expertise. Patel (2011) proposes the OPERA hypothesis with 5 factors that may account for the influence of instrumental music training on brain plasticity and on shared speech processing networks: Overlap in acoustic features in instrumental music and speech; Precision due to the higher demands of music; Emotion, Repetition and focused Attention. Christiner and Reiterer (2013) consider vocal music, singing, as a ‘good indicator of the ability to remember new and unintelligible utterances’ and conclude that the ability to sing improves auditory memory span. In their review of electrophysiological studies of speech segmentation, Schön and François (2011) conclude that musical expertise facilitates the learning of both linguistic and musical structures. Similarly, Schön et al. (2004) and Marques et al. (2007) demonstrate that musical training increased pitch discrimination in both music and language. Most importantly, children who are more sensitive in discriminating sounds due to music training are better on phonological awareness and reading tests (Lamb and Gregory, 1993; Douglas and Willats, 1994; Anvari et al., 2002; Peynircioglu et al., 2002; Bolduc and Montésinos-Gelet, 2005; Gromko, 2005; Forgeard et al., 2008; Moreno et al., 2009; Degé and Schwarzer, 2011; Herrera et al., 2011; Moritz et al., 2012).

Similarly, the four meta-analyses of Butzlaff (2000), Bolduc (2008), Standley (2008), and Lessard and Bolduc (2011) that reviewed more than 70 different multidisciplinary studies also point to a relationship between musical training and reading skills, mainly reading in L1. Butzlaff's meta-analysis reviewed 24 correlational and 6 experimental studies. The author concluded that results strongly and reliably associate music performance with standardized reading/verbal tests but that the causal nature of the relationship remained to be demonstrated. For instance, the influence of a factor such as teachers' expectancy could not be ruled out. Bolduc (2008) reviewed 13 studies and concluded that emergent literacy of preschoolers with or without learning difficulties is affected positively by musical instruction. Standley (2008) reviewed 30 studies related to music-related reading instruction and specific reading skills in order to make pedagogical recommendations about reading failure. The author differentiated two main music education styles underlying these studies: on the one hand, studies including multisensory programs based on Orff, Kodály, or Dalcroze methods that focus on singing, rhythm, instrument playing, or movement to music, and on the other hand, those that rely on extensive practice in choral, band, or orchestral ensembles. Although, the studies in general indicated benefits for reading, the great diversity of intervention programs and of variables such as age and motivation did not allow to draw firm conclusions, except that the younger the child, the stronger the gains from music interventions. According to this analysis, “Music activities that incorporate specific reading skills matched to the needs of children at-risk for reading difficulties (as well as special education, ESOL, early intervention) will enhance reading instruction” (Standley, 2008, p. 29). Finally, Lessard and Bolduc (2011) analyzed 17 studies that added evidence to the link between musical learning and reading among first to third graders. However, causality was not demonstrated due to differences between musical intervention programs, musical, and reading skills, sample sizes and also that many of these studies were unpublished works (doctoral dissertations, master thesis, pilot studies).

Turning to L2 acquisition, Fonseca-Mora and Gómez-Domínguez (in press) reviewed 27 experimental, correlational and quasi-experimental studies on music and language reading published between 2001 and 2013 and concluded that only 7.4% referred to L2 learning, thereby indicating a gap in this field. Marques et al. (2007) showed behavioral and electrophysiological evidence that musical expertise influenced the detection of pitch manipulations on sentence-final words in a foreign language. In this review, Chobert and Besson (2013) proposed that musical training may reduce phonological deficits in second language learning.

From an educational perspective, it remains unclear if the benefits for learning to read in L2 are based on general music instruction or on singing musically-supported phonological input matched to specific reading skills (Standley, 2008, p. 29). In a study with 11-year-old Spanish English Foreign Language (EFL) learners, Toscano-Fuentes and Fonseca-Mora (2012) showed that the use of musical-linguistic activities in the foreign language classroom improved reading skills as well as speaking and listening skills. Herrera et al. (2011) discussed the effects of a phonological and a musical plus phonological training program on the reading readiness of native and L2 Spanish-speaking children and stressed that the musical training approach helped native and foreign Spanish learners to outperform those without musical training in the ability to identify word endings, possibly because children's songs make rhyming words particularly salient (Herrera et al., 2011, p. 78). However, preschoolers who received the phonological training program without musical support obtained better results in phonological awareness and naming speed.

Our concern in this study is based on the fact that poor foreign language readers, in this case Spanish learners of English, lack phonological language skills, phoneme awareness, letter–sound knowledge and rapid automatized naming (Hulme and Snowling, 2014). In addition, the Spanish school curriculum does not include musical training. All second-grade Spanish children who participated in this study were very low-proficiency English language learners with classrooms located in suburban schools. This is important as this implies that there was no initial selection of participants. However, socio-cultural background, reading skills and working memory were assessed before training to ensure that the different groups were homogenous. Learners' musical aptitude was also tested as it has been described as an individual difference in language learning (Slevc and Miyake, 2006).

### Purpose of the current study

Based on previous studies showing that phonological awareness is related to reading abilities and that music training improves phonological processing, the aim of the present study was to test for the efficiency of a new method for teaching to read in a foreign language. Specifically, we aimed at testing the efficacy of phonological training programs, with and without musical support that aimed at improving early reading skills in 7–8-year-old Spanish children learning English as a foreign language (EFL). Of interest was also to explore the impacts of these training programs on working memory and decoding skills. To achieve these goals we tested three groups of children: a control group, an experimental group with non-musical intervention (active control), and an experimental group with musical intervention.

A video was selected in both experimental groups to teach early reading skills such as the alphabetic principle, phonological awareness and phonics. The musical experimental group was taught through video-clips that included musical elements such as songs with lyrics. The non-musical experimental group (or active control group) received the same phonological training program as the musical group but the program did not include melodies. The control group was taught in the traditional way without specific phonological awareness training nor musical support.

We hypothesized that the level of performance would be higher for teaching approaches that included phonological training with or without musical support than for traditional teaching methods. Moreover, we also hypothesized that musical support in a phonological training program for beginner EFL students would be an added value when learning to read because simple, rhythmic and repetitive melodies may induce the song-stuck-in-my-head phenomenon, a rehearsal loop that may improve sub-vocal rehearsal. The songs, created especially for improving phonetic aspects, were characterized by their slow pace and by the simplicity of their melodic contours. They were easy to memorize and, if activated periodically, they could favor automatized decoding. Finally, to determine the effects of the pedagogical intervention, pre/post tests and regression analyses including knowledge of sounds and letters, reading fluency and their interaction with working memory were computed.

## Materials and methods

### Participants

A pre-post comparison design was used to examine training effects. Three second grade classes including 63 students (*X* = 7.6 years old, *SD* = 0.4; 29 boys and 34 girls) were selected from two primary schools located in the same school district. Mean age between the three groups was not significantly different (*F* < 1) nor were the gender differences [χ^2^_(2, 63)_ = 1.97, *p* = 0.374]. At the beginning of the study, the music experimental group (*n* = 18) comprised 8 females and 10 males (mean age: *X* = 7.71, *SD* = 0.40). The non-musical experimental group (*n* = 22) comprised 11 females and 11 males (mean age: *X* = 7.58, *SD* = 0.35) and the control group (*n* = 23) comprised 15 females and 8 males (mean age: *X* = 7.67, *SD* = 0.51).

### Procedure

Prior to the beginning of the study, the school community was informed, organizational aspects were discussed and formal consent was granted. The control group and the non-music experimental group (with phonological training) were located within the same school. The music experimental group was located in a different school to avoid contamination if learners would sing the learned melodies in the playground. Teachers of both experimental groups were trained for several weeks before the start of the experiment. During the 2 weeks prior to the beginning of the training period, trained language graduate assistants and graduate assistants in psychology (supervised by a neuropsychologist and two language researchers) tested the musical abilities, early reading skills, working memory and socio-cultural level of the 63 learners individually in a quiet room at their school. Immediately after the 11-week training period, reading skills and working memory of the young learners were tested again.

### Questionnaires

A battery made up of four questionnaires was used:

- A socio-cultural survey, administered prior to the training program, to identify the main family characteristics and reading habits of the children.
- A musicality test to control for musical aptitudes. This test is an adaption of Hernández-Hernández and Santiago-González (2010) and included items that measured pitch, intensity, duration, rhythm, musical timbre and musical tempo. Two practice trials preceded each item to ensure that children understood the task.
- The *Wechsler Intelligence Scale for Children, 4^th^ Edition* (WISC-IV, Spanish version) standardized neuropsychological assessment pre and post training. Selected tests included Digit Span and Letters and Numbers Sequencing subtests to assess auditory memory span.
- The *Early Grade Reading Assessment* (EGRA) in its English version including:
  ◦ Letter name knowledge: name as many upper and lowercase letters as possible in 1 min. Letter presentation was random.
  ◦ Initial sound identification: identify the initial sound of ten words read aloud by the test administrator.
  ◦ Oral reading fluency: read a dialog with accuracy, speed and fluency in 1 min.

### Training program (experimental groups)

Children in both training programs received two 1-h sessions per week, for a period of 11 weeks and a total of 22 sessions. Video-clips were used in both training programs to help learners attach meaning to the minimal units of discursive articulation. Activities focused on the development of phonological awareness and phonics (e.g., auditory exercises that emphasized alliteration, word-onset awareness, and initial sound identification in frequent English words). Other activities focused on the learning of the alphabet (e.g., English letter-names and letter-sounds). The teacher in the music group used videos supported by songs with subtitles, characterized by simple and repetitive melodies and rhymes (Gértrudix Barrio and Gértrudix Barrio, 2010). Children in this group were trained in song perception and production and they were encouraged to sing the material learned in the hope that the catchy songs would foster self-initiated rehearsal. Children in the non-musical group worked on the same reading skills and contents but through attractive and colorful videos, posters, and audio-books without music. Both teachers planned together and simultaneously their lessons so that they were teaching the same thematic units at the same time.

### Phonological training program

The phonological training program included the following tasks that were supported by visual materials (e.g., posters and flashcards):

#### Practicing with single-letter sounds

Children learned the names and sounds of the letters of the English alphabet. Letter-names and letter-sounds were presented using videos, posters, and audio-books in in the non-musical group, and using songs and subtitles the music group.

To establish a relationship between letters and sounds, frequent one-to-three syllable words were spelled and pronounced at the same time the songs and non-musical videos were played. Every word with a common spelling and phonetic pattern was classified into different word-bank lists written on a board. For example, words with the same middle sound as “book” (/u/ sound:*book, foot, look, food*) or those with the “ph” grapheme (/f/ sound: *elephant, phone, dolphin*) were included in the same list. This task was used for students to automatize graphemes-phonemes matching as well as English pronunciation, structures, and rules. Most of the tasks were designed to foster learners to use their auditory discrimination and production skills, such as:

(a) Onset and rime detection tasks: learners were asked to identify initial and final phonemes in words. For example, “What is the first sound in the word ‘fish’? or ‘What is the last sound in ‘fox’?”

Phonological oddity tasks were also included in which learners were asked to spot the odd word out when listening to three different words, two of them sharing the same initial phoneme (e.g., “which word begins with a different sound: jam, yoghurt, juice?”).

(b) Oral blending skills, manipulation of sounds in words and word formation tasks when learning and reading new words. Learners were required to change the initial sound of a word to create a new word. For example, to change the initial sound of the following words (*hen, hill, hat, hot, hump*) to /p/ or to choose which words could be made with the following initial phonemes: c, b, l, f, v, h, j. Tasks that required learners to change the middle vowel in a word to another that had the same sound to find out the correct spelling that matched a picture presented on a flashcard (“jamper or jumper?,” “mauth or mouth?,” “food or fud?”).

#### Phonics and spelling

Word choice tasks based on spelling were also instructed: learners made words using various combinations of vowel and consonant letter-cards, putting them on a board for all students to see. Sound matching tasks based on blending words onset graphemes and ending phonemes (rimes), using “the phonic wheel,” were to improve learner's spelling skills.

### Traditional program (control group)

The traditional teaching program was based on the idea that phonological decoding skills are learned from direct exposure to foreign language and transfer directly from L1. The teacher used the syllabic and global word approach as classically described in L1 textbooks. The curriculum for teaching English to second graders mainly included vocabulary (numbers, colors, food, animals, parts of the house, verbs), some easy verbal routines (greeting, saying good-bye…) and simple sentences such as “I have/not…, I like/ I don't like… ” Flashcards and games were used to help students to increase motivation for the English lesson.

### Data analysis

One-Way repeated measures Analyses of Variance (ANOVAs) were conducted to test for before training differences in socio-cultural factors and musical aptitude between groups. Moreover, ANOVAs were also computed to test for differences before and after training that included Group (Control, musical, and non-musical) as well as Session (pre vs. post training) as factors. Finally, multiple regression analyses with interactions (Aiken and West, 1991; Rosel et al., 2014) were also conducted to test for the effects of the training program in the experimental and control groups. Data analysis was performed using the 21.0 SPSS statistics package.

To determine whether the intervention produces different effects in the 3 groups (non-musical experimental, musical experimental, and control groups), an ANCOVA was conducted on the differences between groups after training, controlling for the level of performance before training (i.e., “prior knowledge”) for each one of the three tasks. In addition, we computed a regression analysis for each one of the three tasks. Reading variables_pre_, WM_pre_ and the interactions with the Group factor (musical, non-musical, and control) were included.

## Results

### Before training

The three groups were homogeneous regarding their socio-cultural background (see Table 1). Learners' musical aptitude was also homogeneous within the three groups before training, with a normal distribution [Kolmogorov-Smirnov's test: K-S = 0.622, *p* = 0.834 and Levene_(2, 60)_ = 0.495, *p* = 0.612]. However, results revealed significant differences between groups [*F*_(2, 60)_ = 14.175, *p* < 0.001]: mean scores in the non-musical experimental (NME) group (*X* = 27.2, *Sd* = 3.7) were significantly higher than in the control group (Cont: *X* = 22.6, *Sd* = 3.2; Bonferroni = −4.62, *p*<0.01) and in the musical experimental group (ME: *X* = 21.3, *Sd* = 4.4; Bonferroni =−5.89, *p* < 0.01), with no significant differences between the control and musical group (Bonferroni = 1.27, *p* < 0.855).

**Table 1:** **Test for between groups differences on socio-cultural variables**.

	***df***	**X^2^**	***P***
Level of studies (father)	10	10.22	0.42
Level of studies (mother)	10	7.67	0.66
Same language spoken at home and at school	2	0.83	0.66
Home language other than Spanish	2	3.16	0.20
Reading at home besides schoolwork	2	0.79	0.67
Family member who reads more	2	3.99	0.13
Someone reading aloud to participants	2	3.10	0.21
Listening to music	2	0.20	0.90
Frequency of listening to music	6	3.73	0.71

Working memory (WM) data showed a normal distribution (K-S = 0.495, *p* = 0.967), but this was not the case for “Correct Letters read in English” (K-S = 4.069, *p* < 0.001), “Initial Sound Identification” (K-S = 1.021, *p* = 0.021) and “Correct Words Read in a Dialog in English” (K-S = 3.659, *p* < 0.001). Non-significant differences were found between the three groups [*F*_(2, 60)_ = 0.55, *p* = 0.58]. The H non-parametric test of Kruskal-Wallis showed no between-groups differences in the “Correct letters read in English” (H_K-W(2)_ = 2.977, *p* = 0.226) and the “Correct words read in a dialog in English” (H_K-W(2)_ = 5.159, *p* = 0.076) tasks, but significant differences in the “Initial sound identification” task (H_K-W(2)_ = 6.562, *p* = 0.038), with higher scores in ME than in NME group (Md_M_ = 94, Md_N-M_ = 100, 5; U_M-W_ = 110, *p* = 0.016).

In sum, the three groups were similar in terms of socio-cultural background and working memory but the non-musical experimental group had significantly higher musical aptitudes than the other two groups and the musical experimental group showed higher scores in the “Initial sound identification” task than the NME group.

### Before vs. after training comparisons

Results of non-parametric Wilcoxon tests for the variables with non-normal distribution were all significant: the level of performance in “Correct letters read in English” (Z_W_ − 4.791, p. 001), “Correct words read in English dialogs” (Z_W_ − 3.429, p. 001) and “Initial sound identification” (Z_W_ − 3679, p. 001) were higher after than before training. By contrast, results for WM (ANOVAs) were not significant [*F*_(2, 61)_ = 0.001, *p* = 0.974] and neither was the interaction between Session (WMpre vs. WMpost) and Group [ME, NME and Cont; *F*_(2, 62)_ = 1.90, *p* = 0.16]. Thus, we decided to use the results obtained for WM_pre_ to avoid the potential influence of the WISC test on WM_post_.

### “correct letter names read in englisth” task

The main effect of Group was significant after training [*F*_(2, 60)_ = 9.81, *p* < 0.01, η^2^*_p_* = 0.247] with a larger effect when “prior knowledge” was controlled for [*F*_(2, 59)_ = 16.16, *p* < 0.01, η^2^*_p_* = 0.354, β = 0.979], explaining 41.5% of the variance (*R^2^_c_* = 0.415). Planned Bonferroni contrasts revealed that the level of performance increased significantly in both experimental groups compared to the control group (*p* < 0.01, IC_EM-C_ [5.11, 16.56], IC_ENM-C_ [5.67, 16.44], with no differences between the musical and non-musical experimental groups (*p* > 0.05, IC_EM-ENM_ [−5.95, 5.50]).

Table 2 shows the interaction terms between working memory scores in the control group and in the non-musical group through dummy variables, with the musical experimental group as reference. Specifically, the model explained 52.2% variance in number of letters read per minute (*R*^2^ = 0.52). The number of correct letters read was predicted by the combined effect of Group and WM, with significantly lower scores in the control group than in the musical group.

**Table 2:** **Regression coefficients for “correct letters read in English” task after training**.

	**Unstndzed Coeff**	**Std error**.	***t***	**Sig**.
(Constant)	−20,600	11,026	−1868	0.067
Combined punctuation in WM_pre_	0.326	0.115	2832	0.006
Correct letters read_pre_	0.563	0.126	4477	0.000
Dummy non-musical experimental	22,919	13,671	1676	0.099
Dummy control	22,364	13,436	1665	0.102
Interaction WM_pre_—Dummy non-musical group (Ref. G. M. Exp.)	−0.243	0.141	−1728	0.090
Interaction WM_pre_—Dummy control group (Ref. G. M. Exp.)	−0.351	0.141	−2484	0.016

**Figure 1:**
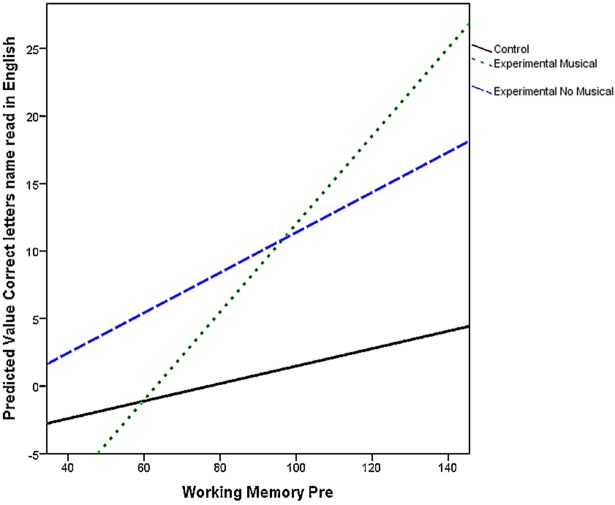
**Working memory and correct letter scores**.

As can be seen on Figure 1, learners with higher WM_pre_ scores before training improved more in this task with larger improvements in the musical group. After training, the difference between the musical and control group was significant with no difference between the experimental (non-musical and musical) groups.

### Correct words read in a dialog in english

The main effect of Group was significant after the intervention [*F*_(2, 60)_ = 5.216, *p* = 0.008, η^2^*_p_* = 0.148, β = 0.812]. However, no pre-post differences were found when “prior knowledge” was controlled for [*F*_(2, 59)_ = 1.018, *p* = 0.368, η^2^*_p_* = 0.034]. The model explained 65.6% of the variance (*R*^2^_c_ = 0.656) with confidence intervals at 95% (Bonferroni tests: IC_CTR-EM_ [−3.22, 5.18], IC_CTR-ENM_ [−5.31, 2.36], IC_EM-ENM_ [−6.82, 1.90]).

As can be seen on Table 3, scores obtained in both groups, NME group (lower initial value) and Cont group (higher initial value), were based on significantly different initial values than in the ME group. Although, the interaction was not significant, there was a trend for the largest increase in this task to be found in the NME group (steeper slope), then in the ME group and the slowest evolution to be found in the Cont group. In this case, *R*^2^ = 0.505 is reached.

**Table 3:** **Regression Coefficients for “correct words read in English” post**.

	**Unstandardized Coefficients**	**Std error**.	***t***	**Sig**.
(Constant)	−8.57	5.69	−1.50	0.14
Combined punctuation in WM_pre_	0.18	0.06	3.23	0.00
Correct words read in a dialog in English_pre_	−0.01	0.01	−0.87	0.39
Dummy non-musical experimental	7.48	2.53	2.96	0.00
Dummy control	5.29	2.49	2.12	0.04

**Figure 2:**
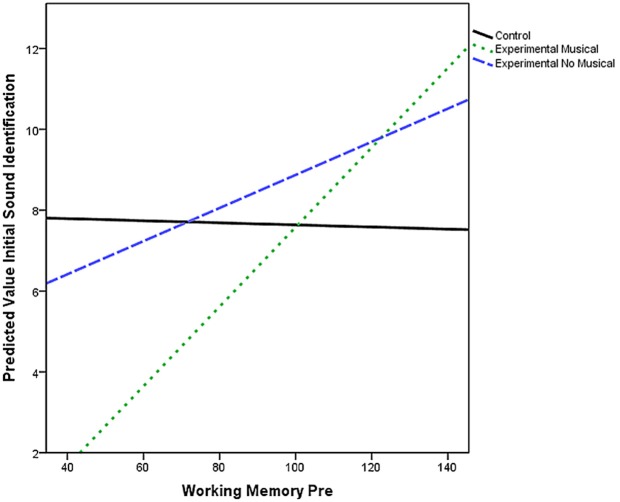
**Working memory and correct words read in a dialog**. The number of correct words read in a dialog contains the significance of the working memory score.

It can be observed (Figure 2) that the non-musical group (lower initial score) and the control group (higher initial score) have significantly different initial values from the musical group. Differences were observed in the intercepts (the value of *Y* when *X* = 0).

### Initial sound identification

After training, the main effect of Group was significant [*F*_(2, 60)_ = 3.352, *p* = 0.042, η^2^_p_ = 0.101, β = 0.612]. However, no pre-post differences were found when “prior knowledge” was controlled for [*F*_(2, 59)_ = 1.47, *p* = 0.602, η^2^_p_ = 0.017]. The model explained 45.3% of the variance (*R*^2c^ = 0.453) with confidence intervals at 95% (Bonferroni tests: IC_CTR-EM_ [−1.30, 1.36], IC_CTR-ENM_ [−1.74, 0.81], IC_EM-ENM_ [−1.90, 0.91]. Significance of the interaction terms between working memory and control group scores contrasted with the musical group on the total scores in this task is reported in Table 4. In this model *R*^2^ = 0.525 is reached.

**Table 4:** **Regression Coefficients for “initial sound identification in English” post**.

	**Unstndized Coeffs**.	**Std error**.	***t***	**Sig**.
(Constant)	−1.16	2.65	−0.44	0.66
Combined puntuation in WM_pre_	0.06	0.03	1.99	0.05
Initial sound identification_pre_	0.50	0.09	5.71	0.00
Dummy non-musical experimental	5.21	3.28	1.59	0.12
Dummy control	7.36	3.24	2.274	0.03
Interaction WM_pre_—Dummy non-musical group (Ref. G. M. Exp)	−0.05	0.03	−1.466	0.15
Interaction WM_pre_—Dummy control group (Ref. G. M. Exp)	−0.08	0.03	−2.289	0.03

**Figure 3:**
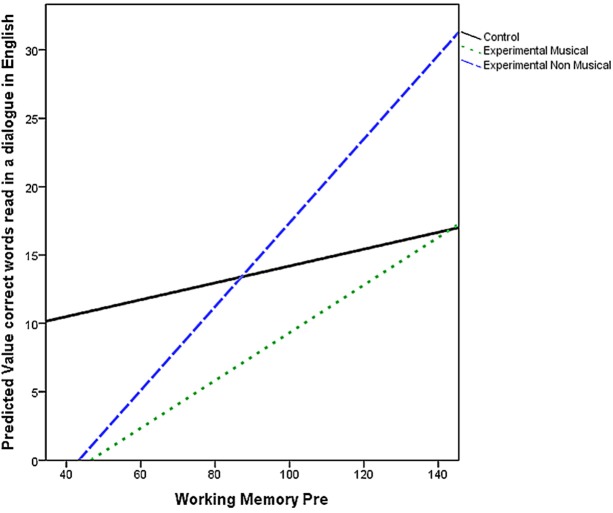
**Working memory and initial sound identification**. Slopes of regression equation for each group when the interaction was considered.

As can be seen on Figure 3, no improvement was found in the control group. By contrast, the level of performance improved in both experimental groups with higher scores in the musical group. The difference between control and musical group was significant with no difference between the musical and non-musical experimental groups.

## Discussion

The main objective of this study was to examine the effects of phonological training programs with and without music support on reading abilities in 7–8 year-old Spanish children learning English as a foreign language. A positive outcome would allow us to propose an alternative, research-based, foreign language teaching method. Most studies point to instrumental musical training as an important factor contributing to reading skills (Anvari et al., 2002; Peynircioglu et al., 2002; Bolduc and Montésinos-Gelet, 2005; Gromko, 2005; Forgeard et al., 2008; Moreno et al., 2009; Degé and Schwarzer, 2011; Herrera et al., 2011; Moritz et al., 2012). However, instrumental musical training is often difficult to implement in primary schools when music classes are not included in the curricula. By contrast, singing is often practiced in kindergarten and early primary schools. Christiner and Reiterer (2013) pointed out that singing is similar to music at the acoustic-perception level and can also help detecting rhythmic cues in foreign languages. Consequently, singing can contribute to improve speech production and is easier to implement. Thus, rather receiving an instrumental music training, the young foreign language learners involved in this experiment benefitted from a phonological training program based on repeatedly singing rhythmic melodies during the 11-weeks training program.

Results of pre vs. post comparisons showed that children in the musical and non-musical (i.e., active control) training groups performed significantly better than children in the control group regarding the “Correct letters read in English” and the “Initial sound identification” tests. Moreover, a trend was found in the “Correct words read in an English dialog” with larger increase in the non-musical group compared to the musical group and smallest increase in the control group. Finally, predictive analyses based on regressions with interactions, and taking working memory into account, indicated no significant differences between musical and non-musical groups, both doing better than the control group. These results clearly point to the beneficial effects of the phonological teaching approach but the further impact of the music support was not demonstrated.

Children in the non-musical group performed higher than children in the musical and control groups in the musicality test presented before training. This was possibly linked to these children being from Spanish gypsy families who typically show strong rhythmic abilities (Gil and Azcune, 2012). In this respect, using a longitudinal approach, David et al. (2007) showed that sensitivity to musical rhythm was related to the ability of decoding complex words requiring the use of linguistic stress and they argued that rhythm predicted reading ability from grade 1 to 5 in primary school. Similarly, Moritz et al. (2012) concluded that the rhythmic abilities students developed when they were preschoolers correlated with their phonological abilities in second grade. Thus, the higher level of performance of children in the non-musical group in both the “correct letters read in English” and the “correct words read in an English dialog” may be linked to English being a stress-timed language (while Spanish is syllable-timed) strongly relying on rhythmic cues. Moreover, early phonetic processing may be organized differently in children with high musical aptitudes, as shown in adults with stronger musical expertise (Ott et al., 2011). In other words, learners with higher musical aptitudes may tend to benefit more from the phonological training program than children with lower musical aptitudes. Importantly, however, while children in the music group showed low musical aptitudes before training, they nevertheless performed better than the control group in the tests described above. Therefore, the phonological training programs with and without music support seem to have significant effects on early reading skills.

Of interest in this study was to examine the influence of working memory and how it interacted with the effects of other factors. Previous reports in the literature have shown that instrumental musical training significantly improved working memory (Ho et al., 2003; Franklin et al., 2008). More recently, Christiner and Reiterer (2013) showed that singing also improved auditory working memory span in Austrian adult singers performing in Hindi. Surprisingly, no such differences between WM_pre_ and WM_post_ were found in the present study. However, as seen in Figure 1, the largest increase in WM scores is found in the music group. Moreover, the higher the scores in WM_pre_, the larger the increase in “correct letters read” scores after training. This effect was larger in the experimental groups than in the control group with no differences between experimental groups. It may be that the speech sounds as well as the visual and orthographical elements, addressing the phonological loop and the visuo-spatial sketchpad, respectively (Baddeley, 2012), used in the two experimental groups, improved learners' basic reading skills. In addition, and in line with Christiner and Reiterer (2013) results, it may be that the repetitive use of melodies with memorable lyrics allowed young learners to better retain verbal materials and relevant foreign speech sounds. Finally, the lowest level of performance in the control group clearly showed that direct transfer from learners' L1 reading skills to another language should not be taken as granted in the foreign language classroom.

One final aspect that deserves comments is the positive impact of the phonological and musical-phonological programs after a relatively short training duration (11 weeks). This finding is in line with previous results showing significant effects of training after 16, 14, and 4 weeks, respectively in Gromko (2005), Moreno and Besson (2006), and Register et al. (2007). In addition, our results are in line with the conclusions from a meta-analysis conducted by Standley (2008) showing that using music activities that matched the specific reading needs of the children was more important than the duration of training (e.g., training of less than 4 weeks (*d* = 0.61) were equally effective than training over an entire school year (0.33, *p* = 0.37). Nevertheless, and in line with previous longitudinal studies over a school year or longer (David et al., 2007; Moreno et al., 2009; Moritz et al., 2012; Chobert et al., 2014), it would be of interest in further experiments to test for the effects of the phonological and phonological plus music training programs on foreign learning abilities when these programs are applied for a longer duration.

## Conclusions

Acquiring good phonological and decoding skills is of uttermost importance for foreign language learners and these abilities are not necessarily directly transferred from L1 knowledge (specifically when L1 and L2 rely on different phonological systems). Nevertheless, these abilities are needed to access lexical content while reading. The phonological training program based on visual support that was used in this study, improved some of the early reading skills in 7–8-year-old Spanish EFL students. Moreover, learners in the phonological plus musical support training program outperformed children in the control group. Thus, simple rhythmic melodies that work as carriers of visual and orthographic perception may stimulate the rehearsal needed for improving specific phonological skills, thereby providing valuable teaching approaches for learning to read in a foreign language.

### Conflict of interest statement

The authors declare that the research was conducted in the absence of any commercial or financial relationships that could be construed as a potential conflict of interest.
